# Spectral Transfer Learning Using Information Geometry for a User-Independent Brain-Computer Interface

**DOI:** 10.3389/fnins.2016.00430

**Published:** 2016-09-22

**Authors:** Nicholas R. Waytowich, Vernon J. Lawhern, Addison W. Bohannon, Kenneth R. Ball, Brent J. Lance

**Affiliations:** ^1^Human Research and Engineering Directorate, US Army Research Laboratory, Aberdeen Proving GroundMD, USA; ^2^Department of Biomedical Engineering, Columbia UniversityNew York, NY, USA; ^3^Department of Computer Science, University of Texas at San AntonioSan Antonio, TX, USA; ^4^Applied Mathematics, Statistics, and Scientific Computation Program, University of Maryland at College ParkCollege Park, MD, USA

**Keywords:** unsupervised learning, ensemble learning, calibration-free BCI, P300, RSVP

## Abstract

Recent advances in signal processing and machine learning techniques have enabled the application of Brain-Computer Interface (BCI) technologies to fields such as medicine, industry, and recreation; however, BCIs still suffer from the requirement of frequent calibration sessions due to the intra- and inter-individual variability of brain-signals, which makes calibration suppression through transfer learning an area of increasing interest for the development of practical BCI systems. In this paper, we present an unsupervised transfer method (spectral transfer using information geometry, STIG), which ranks and combines unlabeled predictions from an ensemble of information geometry classifiers built on data from individual training subjects. The STIG method is validated in both off-line and real-time feedback analysis during a rapid serial visual presentation task (RSVP). For detection of single-trial, event-related potentials (ERPs), the proposed method can significantly outperform existing calibration-free techniques as well as outperform traditional within-subject calibration techniques when limited data is available. This method demonstrates that unsupervised transfer learning for single-trial detection in ERP-based BCIs can be achieved without the requirement of costly training data, representing a step-forward in the overall goal of achieving a practical user-independent BCI system.

## 1. Introduction

Brain-Computer Interfaces (BCIs) are augmentative devices that decode user intent directly from the brain (Wolpaw et al., 2002). Recent advances in signal processing and machine learning techniques have enabled the application of BCI technologies to fields such as medicine, industry, and recreation (Blankertz et al., 2010; Lance et al., 2012; van Erp et al., 2012). Despite the potential of this recent progress, performance decrements due to intra- and inter-individual variability require most BCI systems to employ time-consuming and costly calibration sessions for each new user and session thus decreasing the overall utility of current BCI systems (Johnson et al., 2011). The use of advanced signal processing and machine learning techniques to minimize or eliminate this need for calibration data is an area of on-going interest for the development of practical BCI systems (Lotte, 2015).

Information geometry is one of many promising techniques for robust brain signal detection and classification. The overarching idea being to map the signal into a Riemannian manifold, such as the cone of symmetric positive definite matrices, in which non-Euclidean statistical inference can be performed (Harandi et al., 2012; Jayasumana et al., 2015). With the development of a computational framework for statistical inference (Pennec et al., 2006), information geometry has been successfully applied to radar signal processing (Barbaresco, 2008), diffusion tensor imaging (Fletcher and Joshi, 2004), and computer vision (Tuzel et al., 2007). Recently, information geometry has been applied successfully to BCI paradigms such as motor imagery (Barachant et al., 2012, 2013) and event related potential (Barachant and Congedo, 2014).

In parallel to this development, advanced machine learning methods such as active learning and transfer learning have sought to reduce or eliminate the need for calibration in BCI systems. Transfer learning, for example, addresses how models and parameters can be used in domains for which they were not initially learned, enabling robust models with little or no new training data. Although there has been much recent work in BCI transfer (see Lotte, 2015) very few general purpose methods for zero-calibration BCI currently exist in the literature. Two successful implementations require task-specific structure and do not generalize to other paradigms. Kindermans et al. achieve zero-calibration BCI on a P300 Speller using a language model to simultaneously inform a traditional Bayesian inference classifier, but this method does not generalize to paradigms which do not lend themselves to hidden Markov process modeling or multiple trial classification (Kindermans et al., 2014). Additionally, zero-calibration methods for Steady-State Visually Evoked Potential (SSVEP) BCIs have been developed that correlate multichannel EEG with sinusoidal reference signals using Canonical Correlation Analysis (CCA) (Bin et al., 2009); however, the CCA method is mainly utilized for SSVEP BCIs due to the well-characterized steady-state frequency response and thus does not generalize well to other BCIs.

A recent review categorizes general purpose machine learning approaches to address calibration in BCI systems as either zero-calibration (methods that utilize zero prior training data) or calibration reduction (methods that utilize a limited or reduced amount of training data) (Lotte, 2015). Zero-calibration methods utilize existing models built from previously collected data to classify new data. Thus, zero-calibration methods are able to immediately classify data from a new subject without requiring a calibration session to collect labeled training data. Zero-calibration methods are further classified as either ensemble or pooled methods based on how they use existing training data. Ensemble methods group data into session and subject-specific classifiers which can make independent classification on a new test subject. These classifiers are treated as bases for classifying a new subject, and thus methods differ in how the independent classifications are combined. Fazli et al. presented a zero-calibration ensemble method which uses an ℓ_2_ regression with an ℓ_1_ penalty to enforce sparsity in the linear combination of ensemble classifiers (Fazli et al., 2011). This method is shown to out-perform an unweighted sum of the classifiers. Pooled methods combine all existing data in a single training set and learn a classification model from the aggregated data, and Lotte et al. compared various zero-calibration pooled methods in Lotte et al. (2009).

Unlike the zero-calibration methods, there has been more progress in calibration reduction with the development of machine learning approaches that utilize minimal training data. These calibration reduction techniques achieve performance comparable with conventional calibration techniques. Lotte and Guan demonstrated P300 Speller classification with minimal calibration data by simply applying regularization to a common calibration-based BCI algorithm (Lotte and Guan, 2009). Metzen et al. present a user-to-user ensemble method in which they augment the method from Fazli et al. (2011) with calibration data (Metzen et al., 2011). Dalhoumi et al. (2015) propose an ensemble method which weights the individual classifiers according to their performance accuracy on a small set of calibration data (Dalhoumi et al., 2014, 2015), and this method is shown to out-perform another recent ensemble transfer method from Tu and Sun which uses not only a static ensemble of classifiers but also adapts a dynamic ensemble based on calibration data (Tu and Sun, 2012). In a pooled transfer approach, Jayaram et al. used a multi-task learning method to learn Gaussian priors to estimate optimal ensemble weights for classification (Jayaram et al., 2016). While all of these approaches offer improvements in the field of BCI transfer learning, all still require the use of calibration data, and those that do not are either limited to a specific BCI paradigm or suffer from large performance decreases.

This paper aims to eliminate the need for calibration and develop a user-independent BCI by proposing a new method, Spectral Transfer with Information Geometry (STIG), which leverages an ensemble of information geometric classifiers coupled with spectral-meta learning (SML), an unsupervised ensemble method for inferring the appropriate weights for a linear combination of classifiers (Barachant et al., 2012; Parisi et al., 2014). The STIG method uses an ensemble of subjects trained with Minimum Distance to Riemannian Mean classifiers (MDRM) to make predictions on the test data of a new subject which are linearly combined with spectral meta-learning. We compare competing methods and show that the STIG method out-performs existing zero-calibration methods such as unweighted sum or ℓ_1_-regularized regression as well as several recent calibration reduction methods with limited calibration data. The STIG method is then validated in an on-line rapid serial visual presentation (RSVP) experiment with single-trial ERP detection where real-time feedback is given to the user.

## 2. Unsupervised ensemble transfer learning

STIG is a real-time, unsupervised ensemble transfer method which dynamically combines independent models based on information geometry to classify streaming data for BCI applications. For our application, we have paired the spectral ensemble method introduced in Parisi et al. (2014) with an information geometric classifier, MDRM, based on its superior performance on ERP-based BCIs (Barachant and Congedo, 2014), but this framework could work with any feature extraction and classification method. Off-line, models are trained using existing data from different subjects and sessions. These models make real-time predictions on the trials of a new subject, and the decisions of each model are dynamically combined according to the inferred accuracy of each model.

### 2.1. Classifier

MDRM learns a map, *f* : *X*→*Y*, which assigns a target or non-target label, *y* ∈ {−1, 1}, to each EEG epoch, **X** ∈ ℝ^*C*×*N*^ (*C* channels and *N* time samples). The classification works in feature space and thus, MDRM comprises a feature extraction step, a map *H* : *X* → Σ, and a classification step, a map *G* : Σ → *Y*, such that the overall classification algorithm is the composition of feature extraction and classification, *f* = *G* ∘ *H*.

#### 2.1.1. Information geometric features of single trial P300 ERP

Let $S={\left\{\left({\text{X}}_{i},y_{i}\right)\right\}}_{i\;=\;1}^{n}$ be a labeled training set of *n* trials for a single subject. Define an extended trial,

(1) $$\begin{array}{l} {\tilde{\text{X}}}_{i}=\left[\begin{array}{c} P\\ {\text{X}}_{i} \end{array}\right]=\left[{\tilde{\text{x}}}_{i}^{\left(1\right)}|{\tilde{x}}_{i}^{\left(2\right)}|\ldots {\tilde{x}}_{i}^{\left(N\right)}\right], \end{array}$$

in which **P** = *E* [**X**|*y* = 1] is a prototypical target response. The covariance across time of the extended trial yields the Extended Sample Covariance Matrix (ESCM):

(2) $$\begin{array}{l} \Sigma_{i}=\frac{1}{N-1}\overset{N}{\underset{k\;=\;1}{\sum }}\left({\tilde{\text{x}}}_{i}^{\left(k\right)}-{\hat{\mu }}_{\tilde{\text{x}}}\right){\left({\tilde{\text{x}}}_{i}^{\left(k\right)}-{\hat{\mu }}_{\tilde{\text{x}}}\right)}^{T}, \end{array}$$

where *T* is the usual matrix transpose and ${\hat{\mu }}_{\tilde{\text{x}}}=E\left[\tilde{\text{x}}\right]$ is the sample extended trial column mean. This signal processing map is defined by the prototypical target response, *H* = *H*(**P**).

#### 2.1.2. Minimum distance to riemannian mean

The Minimum Distance to Riemannian Mean classification algorithm (MDRM) assigns each trial to the nearest class mean. With the ESCM, embedded in a differentiable manifold, the cone of symmetric positive definite matrices, $S_{+}\subset {\mathbb{R}}^{2C\times 2C}$, we can induce a notion of distance by selecting a metric in the associated tangent space (Moakher and Batchelor, 2006; Pennec et al., 2006). As in Barachant and Congedo (2014), we will use the Affine Invariant Riemannian Metric (AIRM) to define distance between any two ESCM:

(3) $$\begin{array}{l} \delta \left(\Sigma_{i},\Sigma_{j}\right)=||\text{log}\left(\Sigma_{i}^{-1/2}\Sigma_{j}\Sigma_{i}^{-1/2}\right)|{|}_{F}, \end{array}$$

where *F* indicates the Frobenius norm. Since a covariance matrix is a translation-invariant transformation, the AIRM, which is invariant to linear transformations, provides an invariant measure of distance over all affine transformations of EEG epochs. This facilitates the robustness of the learning algorithm to rotations, scaling, and translations. With this distance function, the geometric mean of each class, ω_ℓ_, on *S*_+_ is

(4) $$\begin{array}{l} \Sigma^{\left(\ell \right)}=\underset{\Sigma \in {\text{S}}_{+}}{arg min}\underset{i\in I_{\ell }}{\sum }\delta^{2}\left(\Sigma ,\Sigma_{i}\right), \end{array}$$

where *I*_ℓ_ = {*i* ∈ 1, …, *n*|*y*_*i*_ = ω_ℓ_}. Therefore, each ESCM, **Σ**, can be classified according to a mapping defined by its class means *G* = *G*(**Σ**^(−1)^, **Σ**^(+1)^):

(5) $$\begin{array}{l} g\left(\Sigma \right)=\underset{\ell \in \left\{-1,1\right\}}{arg min}\delta \left(\Sigma ,\Sigma^{\left(\ell \right)}\right). \end{array}$$

### 2.2. Unsupervised ensemble learning

As introduced in Parisi et al. (2014), SML provides an unsupervised method to combine the classification decisions of independent models to classify a previously collected and un-labeled data set. It attempts to weight each classifier's decision according to the inferred accuracy of the direct classification. Here, it is applied dynamically to streaming data for BCI applications where the independent models are trained with existing data.

#### 2.2.1. Spectral transfer

Let ${\left\{f_{i}\right\}}_{i\;=\;1}^{m}$ be an ensemble of *m* conditionally independent classifiers trained on *m* different training subjects and ${\left\{{\text{X}}_{j}\right\}}_{j\;=\;1}^{n}$ be independent and identically distributed test trials for classification. Then, $F\left({\text{X}}_{j}\right)={\left(f_{1}\left({\text{X}}_{j}\right),f_{2}\left({\text{X}}_{j}\right),\ldots ,f_{m}\left({\text{X}}_{j}\right)\right)}^{T}$. Also, define the balanced accuracy of classifier *i*, $\pi_{i}=\frac{1}{2}\left(\psi_{i}+\eta_{i}\right)$, which accounts for class imbalance by giving equal weight to sensitivity, ψ, and specificity, η. The sample covariance matrix, **Q** ∈ ℝ^*m*×*m*^,

(6) $$\begin{array}{l} \text{Q}=\frac{1}{n-1}\overset{n}{\underset{j\;=\;1}{\sum }}\left(F\left({\text{X}}_{j}\right)-E\left[F\left(\text{X}\right)\right]\right){\left(F\left({\text{X}}_{j}\right)-E\left[F\left(\text{X}\right)\right]\right)}^{T}, \end{array}$$

approximates a rank-one matrix, **Q** ≈ λ**vv**^*T*^, for which *v*_*i*_ ∝ (2π_*i*_ − 1) (Parisi et al., 2014). This implies that the entries of the principal eigenvector of **Q** will be proportional to the balanced accuracy of the associated classifier.

Under a Maximum Likelihood Estimation (MLE), the most likely label, ŷ, for a trial, **X**, is then

(7) $$\begin{array}{l} \hat{y}=\text{sign}(\sum_{i\;=\;1}^{m}f_{i}(X)(\text{log}\left(\frac{\psi_{i}\eta_{i}}{\left(1-\psi_{i})(1-\eta_{i}\right)}\right)\\ \;+\text{log}\left(\frac{\psi_{i}(1-\psi_{i})}{\eta_{i}(1-\eta_{i})}\right))), \end{array}$$

This provides a natural Expectation-Maximization (EM) extension (Dawid and Skene, 1979). The expectation step consists of calculating the respective sensitivity, ψ = *P*(*f*(**X**) = 1|*y* = 1), and specificity, η = *P*(*f*(**X**) = −1|*y* = −1) of each classifier with the MLE estimates of Equation (7). The maximization step then re-calculates the MLE estimates according to Equation (7). For a first order solution, and/or an initialization of EM, a Taylor Series approximation about (ψ, η) = (1/2, 1/2) yields

(8) $$\begin{array}{l} ŷ\approx \text{sign}\left(\overset{m}{\underset{i\;=\;1}{\sum }}f_{i}\left(\text{X}\right)\cdot v_{i}\right). \end{array}$$

#### 2.2.2. Real-time implementation

For streaming data, the weights of the ensemble classifiers can be dynamically updated with the addition of each trial. Naively, **Q** can be updated according to Equation (6) with each new trial, or for efficient on-line implementation, just **v** can be updated according to the rank-one update to **Q** of a new trial (Gu and Eisenstat, 1994). See Algorithm 1 for the real-time implementation of STIG.

**Algorithm 1:**
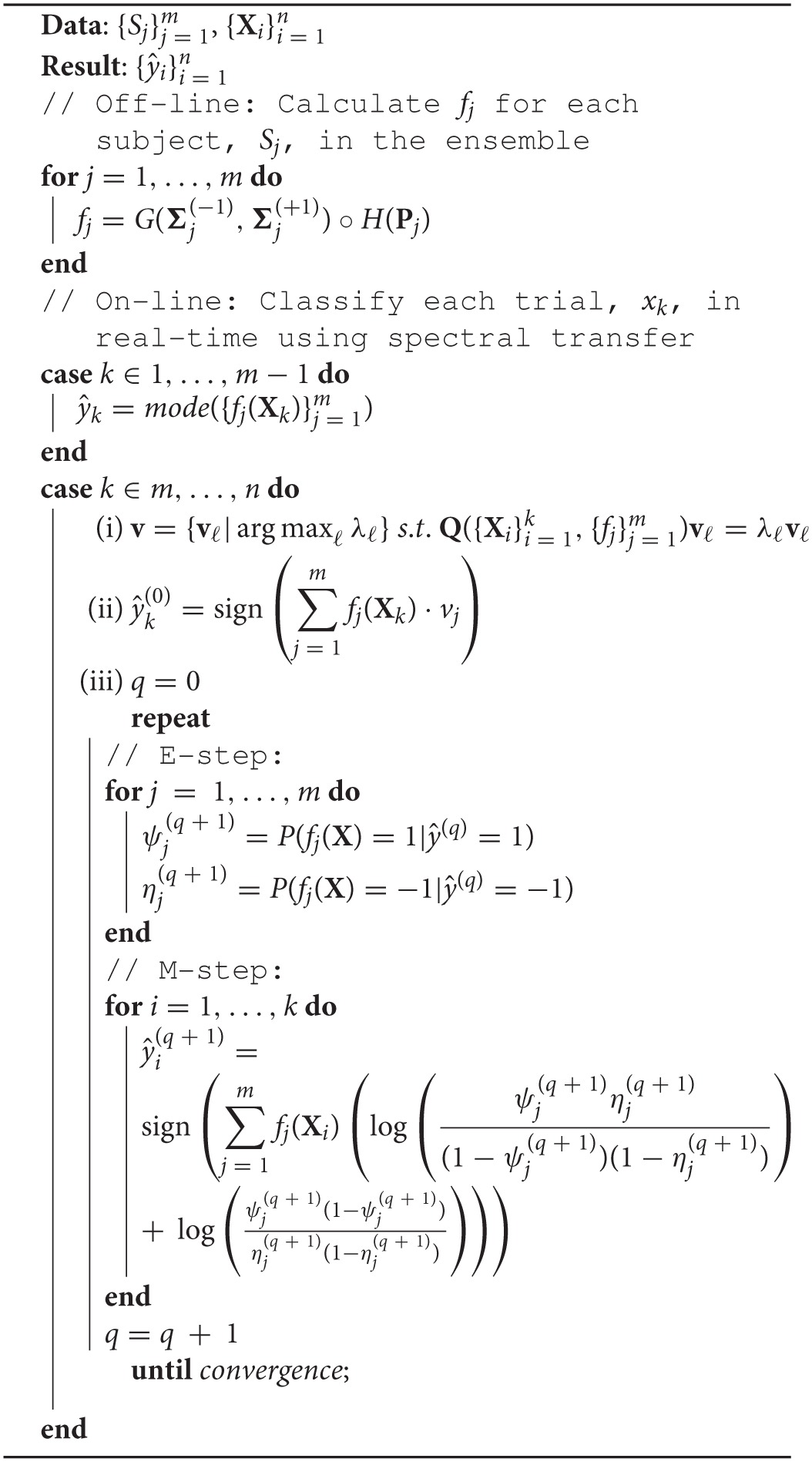
Spectral Transfer with Information Geometry (STIG).

## 3. Experiments

For all experiments, the voluntary, fully informed, written consent of the persons used in this research was obtained as required by federal and Army regulations (U.S. Department of Defense Office of the Secretary of Defense, 1999; U.S. Department of the Army, 1990). The investigators adhered to Army policies for the protection of human subject (U.S. Department of the Army, 1990). All human subjects testing was approved by the Institutional Review Board of the United States Army Research Laboratory.

### 3.1. Rapid serial visual presentation

RSVP experiments leverage the oddball paradigm to elicit a well-characterized P300 visually evoked neural response. In BCI uses, images are shown to a user at a high rate of speed (2–10 Hz), and the user is instructed to anticipate a pre-defined target class which appears infrequently in the images (Gerson et al., 2005; Sajda et al., 2010). When the target class image appears, the resultant P300 response provides a discernible signal for recognition by machine learning methods, allowing a BCI system to discriminate between target and non-target.

### 3.2. Experiment 1: off-line

The data sets used in Experiment 1 were recorded and analyzed previously; a brief description of the original experiment and collection is given here.

#### 3.2.1. Paradigm

**Figure 1:**
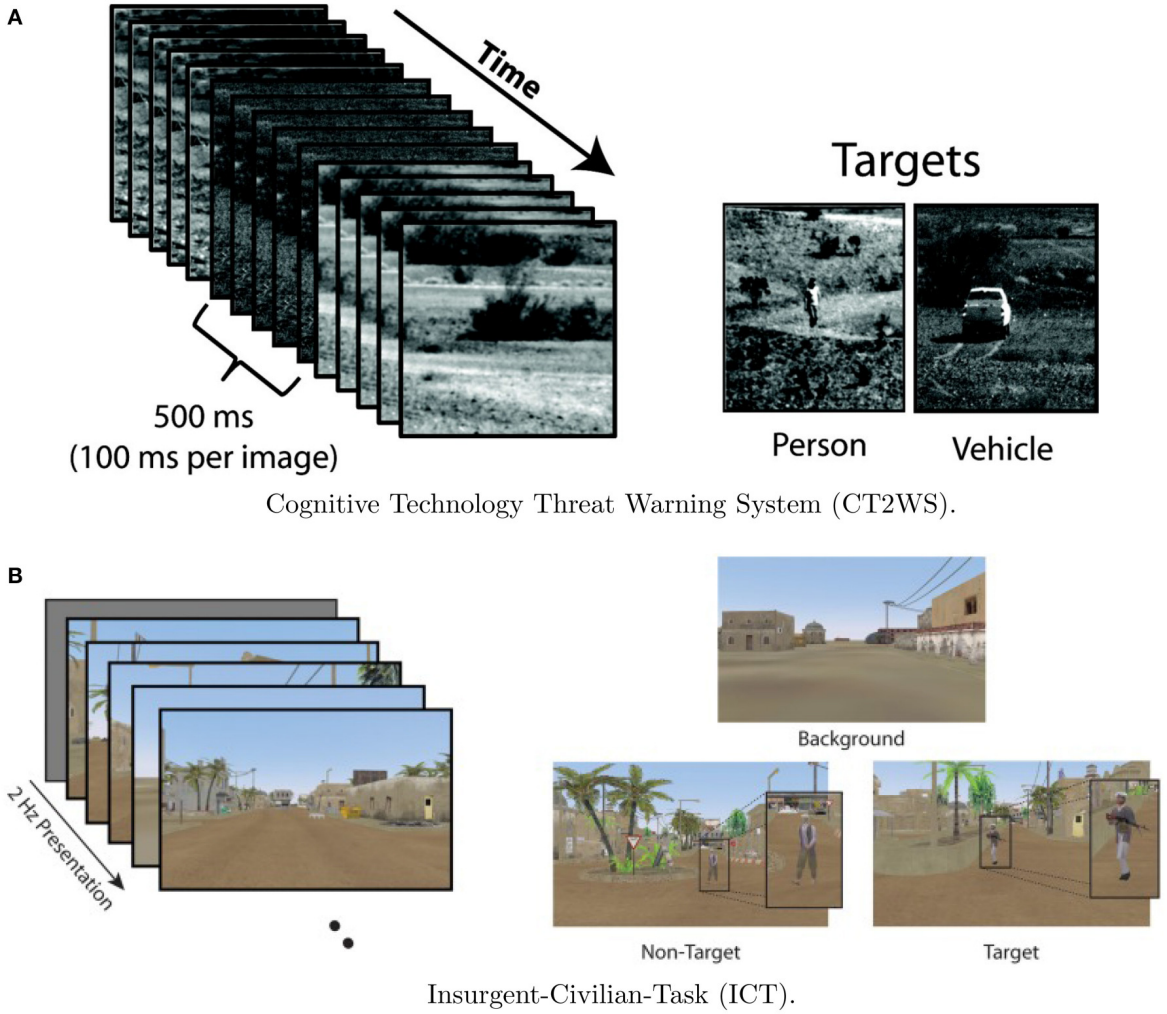
**Visualization of the two RSVP experiments**. Although CT2WS used 500 ms video clips, both experiments effectively implemented 2 Hz RSVP. Example classes for each experiment are shown. Figures reproduced from Ries and Larkin (2013) and Marathe et al. (2015) with permission. **(A)** Cognitive Technology Threat Warning System (CT2WS). **(B)** Insurgent-Civilian-Task (ICT).

Data from Experiment 1 is comprised of two previously collected datasets. The first dataset utilized EEG data from the Cognitive Technology Threat Warning System (CT2WS) DARPA project in which subjects participated in a RSVP task comprised of short movie clips of five images at 10 Hz rate with no inter-stimulus interval (ISI) such that the effective rate between successive movie clips was 2 Hz. Targets consisted of either a person or a vehicle in natural settings. See Figure 1A for a visualization. The target class to background ratio was 1:9. Participants were instructed to manually press a button with their dominant hand when a target movie clip was shown. Participants conducted 25 2-min blocks with a short break between each block (Ries and Larkin, 2013).

For the second data set, Insurgent-Civilian-Task (ICT), subjects participated in a RSVP task with still images of a simulated desert metropolitan environment. Target images contained a person carrying a weapon. Non-target distractor images contained a person without a weapon, and background images contained neither. There were two experimental conditions: Target Only (TO) and Target-Non-target (TN). In the TO condition, only target images and background images were shown. In the TN condition, non-target distractor images were shown in addition to target and background images. Images were shown at 2 Hz with no ISI. See Figure 1B for a visualization. In the TO condition, the ratio of target to background class images was 1:20, and for the TN condition, the ratio of target to distractor to background images was 1:1:14. Participants were instructed to manually press a button with their dominant hand when a target image was shown. Participants conducted six 2-min blocks of the TO condition and six 2-min blocks of the TN condition with a self-paced rest between each block (Marathe et al., 2015). Although there were three stimuli types (target, non-target, and distractor), data from only the target and non-target types were used in the present analysis.

#### 3.2.2. Data collection

Fifteen subjects (9 male, average age 39.5 years) participated in CT2WS. EEG data was collected with a BioSemi Active Two system (Amsterdam, Netherlands) at 512 Hz from 64 scalp electrodes arranged in a 10–10 montage and referenced off-line to the average of the left and right earlobes. All signals were digitally filtered from 0.1 to 55 Hz using an FIR filter. Data was collected using E-Prime software (Psychology Software Tools, Pittsburgh, PA; Ries and Larkin, 2013).

Seventeen different subjects (14 male, average age 34.9 years) participated in ICT. EEG data was collected with a BioSemi Active Two system (Amsterdam, Netherlands) at 1024 Hz from 64 scalp electrodes arranged in a 10–10 montage and referenced off-line to the average of the left and right earlobes. All signals were digitally filtered with an FIR filter from 0.1 to 50 Hz. Data was collected using E-Prime software (Psychology Software Tools, Pittsburgh, PA; Marathe et al., 2015).

#### 3.2.3. Analysis

For single trial classification, all data was high-pass filtered at 1 Hz, down-sampled to 256 Hz, and Independent Component Analysis was performed in which eye movement and muscle activity were manually identified and removed. The data was divided into 1 s epochs time-locked to the stimulus onset, [0, 1*s*], and 18 channels (Fz, Cz, C3, C4, CPz, Pz, P3, P4, P7, P8, POz, PO3, PO4, PO7, PO8, Oz, O1, O2) were selected for classification according to Krusienski et al. (2008), **X** ∈ ℝ^18 × 256^. Whereas, Krusienski et al. (2008) used 19 channels, our electrode montage did not have FCz and therefore only 18 of the 19 channels from Krusienski et al. (2008) were used. For each of the 17 IC subjects, all distractor trials were removed (i.e., trials with a civilian shown). Pre-processing was performed with EEGLAB (Delorme and Makeig, 2004).

For Experiment 1, subject data from both paradigms were combined for a total of 32 subjects. Off-line analysis of the transfer method was conducted by leave-one-subject-out cross-validation. First, a subject-specific MDRM classifier was trained on each of the 32 subjects. For a test subject, all 31 other subject-specific MDRM classifiers comprised the STIG ensemble.

We compared our method against several other calibration suppression and calibration reduction methods which include both ensemble and pooled methods. For all ensemble methods, we use the MDRM classifier as the base classifier, and transfer information in decision space, {−1, 1}. For all pooled methods, we combine information at the feature level using information geometric features. First, we considered the following alternative methods which do not require a dedicated calibration session:

- Max Subject-Specific Classifier (MSS)—We first compared our method against the best direct subject-subject transfer of all 31 possible subjects. Although not possible to implement in reality, it serves as a hypothetical ceiling if we had omniscient knowledge of the available training subjects and could simply pick the best subject-specific classifier for subject-to-subject transfer.
- Majority Vote (MV)—This method uses a simple uniform weighted average of classifications from the ensemble of classifiers. By combining in decision space, an un-weighted linear combination is equivalent to a majority vote.
- ℓ_1_-Regularized Cross-Validation (L1)—This method pools all available training data and learns a weighted linear combination of classifier decisions according to a ℓ_2_ regression with a ℓ_1_ penalty (Fazli et al., 2011). We implemented this method using the MDRM classifier.
- Pooled MDRM (PMDRM)—We implemented a pooled version of the MDRM method in which we combined all available training data into a single MDRM classifier.

Additionally, we compared our STIG method to several of the most recent and highest-performing calibration reduction methods in the BCI literature. Unlike zero-calibration methods, calibration reduction methods require a dedicated calibration session. Thus, to compare against our zero-calibration method, we tested each calibration reduction method with varying amounts of calibration data. As with the zero-calibration methods, we used the MDRM as the base classifier for all calibration reduction methods.

- Accuracy Weighted Ensemble (AWE)—We used the weighted linear combination approach from Dalhoumi et al. (2014), Dalhoumi et al. (2015) to linearly combine classifiers to minimize a Mean-Squared Error (MSE) loss function on a set of calibration data.
- Multi-Task Learning (MT)—This method treats classification as a linear regression problem where feature weights are modeled as a multivariate Gaussian distribution whose unknown mean and co-variance matrix are jointly estimated from multiple training subjects (Jayaram et al., 2016). Once these Gaussian priors are learned, calibration data from the test subject can be used to estimate optimal weights for classification. Since this method utilizes linear regression for classification, it is not possible to implement the MDRM classifier directly. Instead, in an effort to achieve consistency with the other methods, we implement the MT method with the same information geometric features that are used in the MDRM classifier.
- Within-Subject Calibration (CALIB)—This is the standard within subject calibration method that is traditionally used in the BCI literature. Data from the test subject is split into training and testing. In this case, an MDRM classifier, which is constructed using training data from a test subject, is used to classify the remaining test data from the same subject. This traditional classification scheme of calibrating the algorithm to the unique subject during a single session provides the performance ceiling of any calibration reduction or suppression method.

Due to the inherent class imbalance of the RSVP task, the performance of all implemented methods are reported in terms of the balanced accuracy, $\pi =\frac{1}{2}\left(\psi +\eta \right)$, which equally weights accuracy on both target and non-target trials.

#### 3.2.4. Results

##### 3.2.4.1. Comparison of zero-calibration algorithms

**Figure 2:**
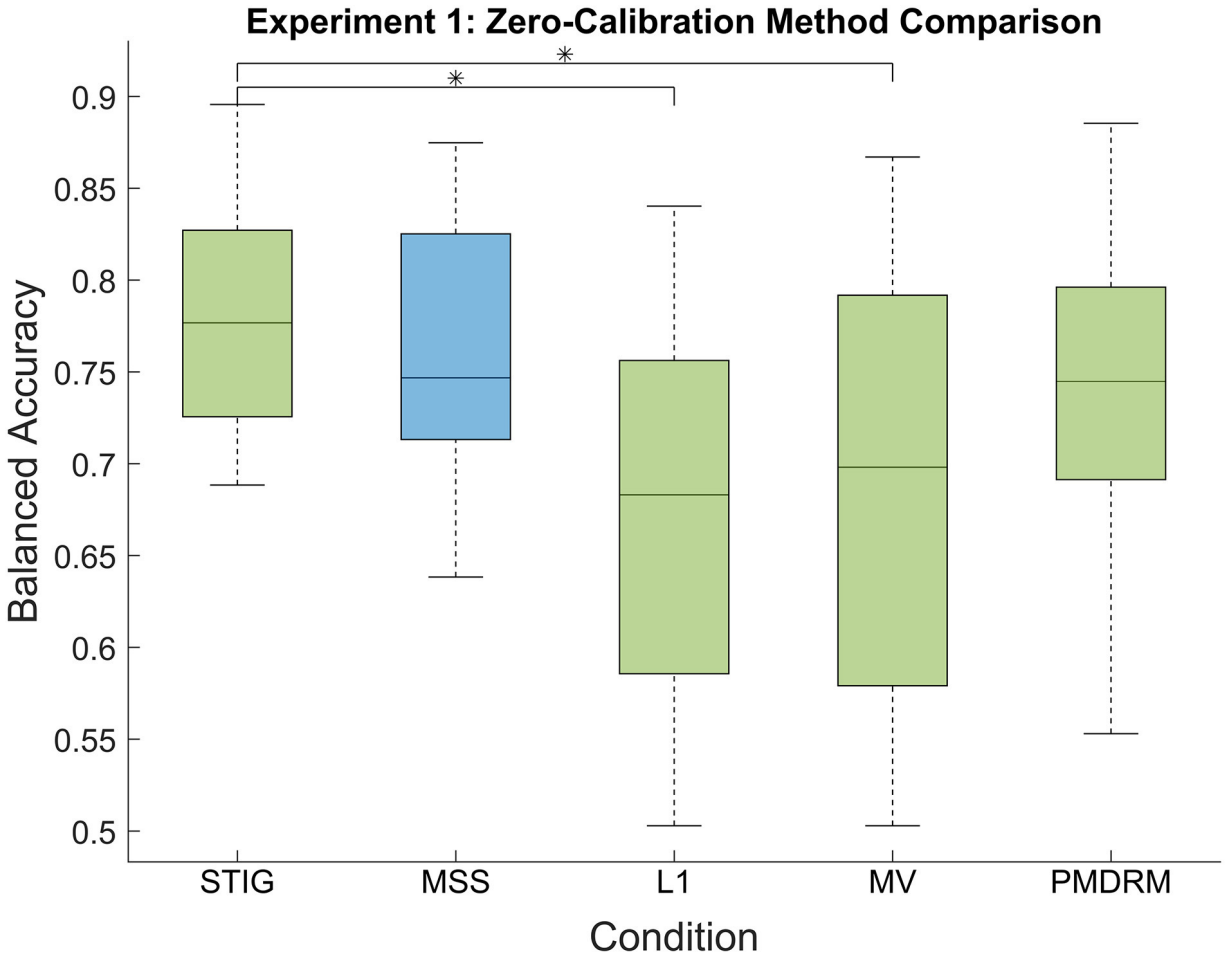
**Balanced accuracy results of the zero-calibration methods for Experiment 1**. Green box plots represent implementable algorithms, while the blue box plot, MSS, represents a method which requires omniscient knowledge. The middle line and boundaries of the box represent median and inner quartile range. The dashed line represents the range, and the asterisks indicate statistical significance of pairwise comparisons between STIG and the competing methods. Here, the STIG method significantly outperforms the L1 and MV methods.

Figure 2 presents the balanced accuracy of each zero-calibration method averaged across all subjects in Experiment 1. A one-way ANOVA showed a significant difference between the five methods tested [*F*_(4, 159)_ = 9.73, *p* < 0.001]. Multiple comparisons were performed for each pair using the Tukey-Kramer method. The proposed STIG method (0.78 ± 0.07) (mean ± standard deviation) achieved comparable performances with the MSS classifier (0.77 ± 0.07, *p* = 0.97) and significantly outperformed the L1 (0.67 ± 0.10, *p* < 0.001) and MV (0.69 ± 0.11, *p* < 0.001) classifiers, illustrating a benefit to be gained from the spectral-based ensemble learner over the competing zero-calibration methods. Additionally, The STIG outperformed our own implemented PMDRM classifier (0.74 ± 0.08), although not significantly (*p* = 0.39).

##### 3.2.4.2. Comparison of calibration reduction algorithms

**Figure 3:**
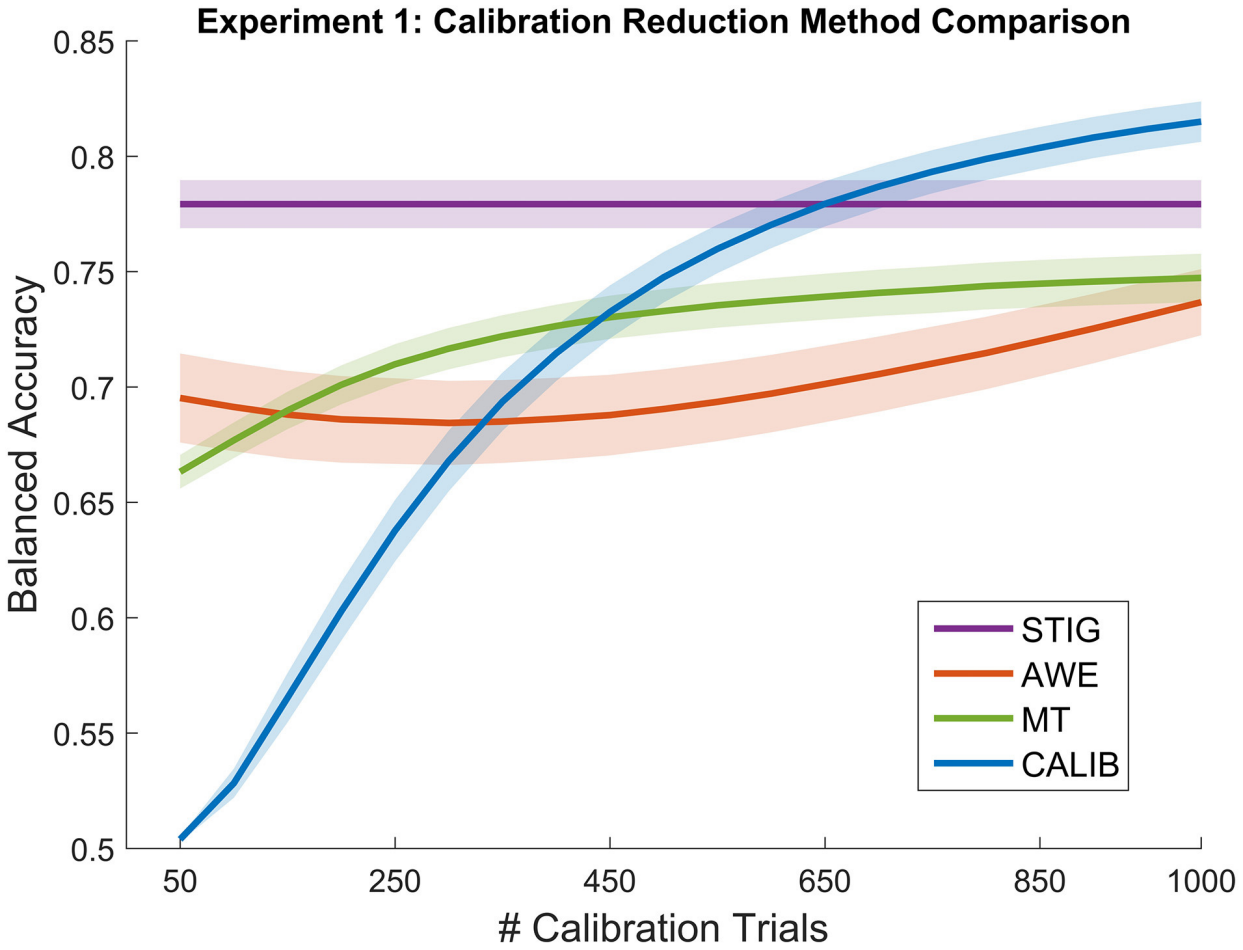
**Balanced Accuracy as a function of number of calibration trials for the calibration reduction methods (AWE, MT, and CALIB) tested in Experiment 1**. The proposed zero-calibration method, STIG, which is independent of the number of calibration trials, is plotted for comparison. Lines represent means averaged across all subjects (*N* = 32) while shaded regions represent standard error.

Figure 3 shows the performance of the STIG method compared to the results of the calibration reductions methods (AWE, MT, and CALIB) as a function of available trials. For testing, the calculation of balanced accuracy for each methods was performed on a fixed number of hold-out trials (600). Each of the calibration reduction methods trained on an increasing number of trials (50–1000 trials at 50 trial intervals). Five hundred bootstrap iterations were performed in which training and testing trials were drawn randomly for each iteration to provide a more robust estimate of the performance distribution. STIG was applied to the sampled testing set in its entirety but did not incorporate any training trials. As shown in Figure 3, each of the calibration-based methods shows a general increase in performance for increasing number of training trials used in the classification.

Statistical analysis was performed between the four methods shown in Figure 3 in a similar fashion as described in the previous section. Since additional ANOVAS were performed for each of the 20 calibration size conditions, the resulting *p*-values were corrected using the false-discovery rate (Benjamini and Yekutieli, 2001). Results show that the STIG method significantly outperforms the AWE method across all numbers of calibration trials used (*p* < 0.05). The STIG method significantly outperforms the MT for the 50–400 calibration trial conditions (*p* < 0.05), and achieves comparable performances for the 450–1000 calibration trial conditions. Similarly, when compared to the traditional within-subject calibration method, STIG significantly outperforms CALIB when 400 calibration trials or less are available (*p* < 0.05). From 450 to 1000 calibration trials, STIG achieves statistically similar performance to the CALIB method (*p* > 0.05).

##### 3.2.4.3. Effect of ensemble size on transfer accuracy

**Figure 4:**
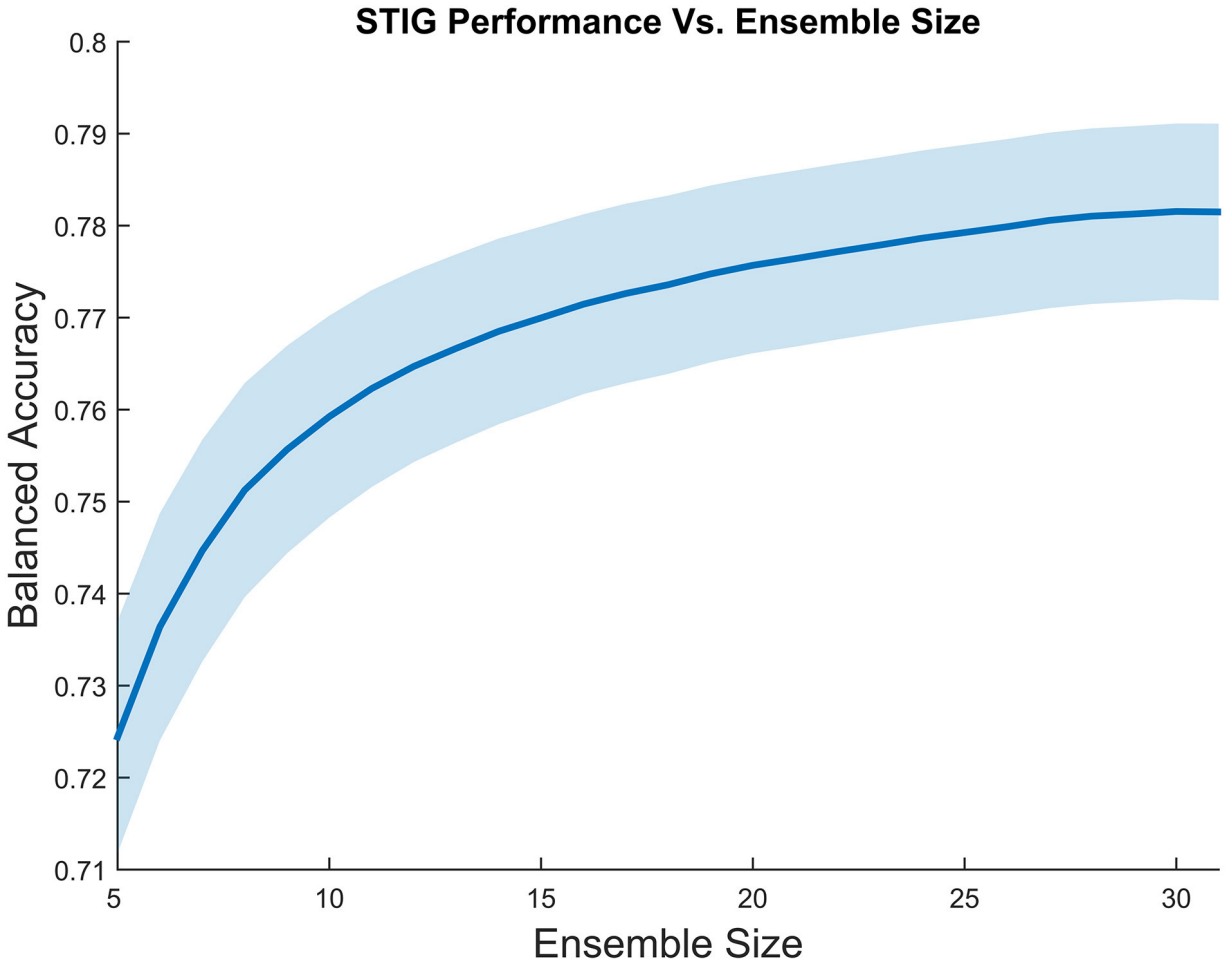
**Grand average balanced accuracy as a function of ensemble size for the STIG method**. The balanced accuracy averaged over 1000 bootstrap runs is reported for each ensemble size. The ensemble size represents the number of training subjects selected for ensemble learning. The shaded region represents standard error.

An obvious question that can be asked about the effectiveness of STIG is the effect of ensemble size on the accuracy of the transfer. Figure 4 shows the effect on performance when various numbers of training subjects are included in the STIG method. For each of the 32 subjects in Experiment 1, 500 bootstrap runs were conducted for each ensemble size (5–31) in which a subset of training subjects were randomly chosen for the ensemble. Figure 4 shows that the performance of the STIG method asymptotically increases with increasing ensemble size.

##### 3.2.4.4. STIG estimation of subject-subject transfer accuracy

**Figure 5:**
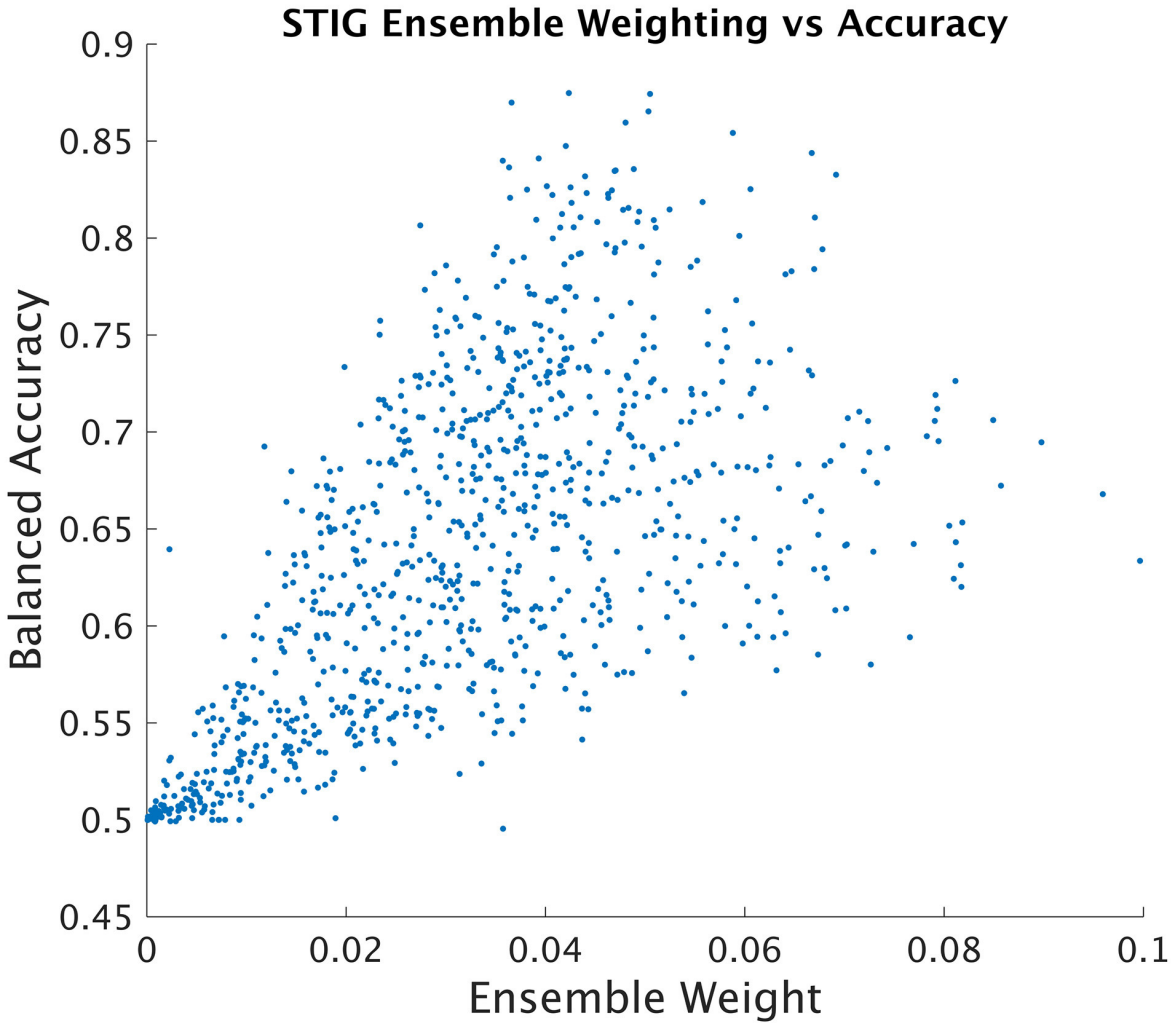
**STIG ensemble weight vs. Subject-Subject Transfer Accuracy**. Each point represents an ensemble training subject whose position on the horizontal axis represents the weight assigned by the STIG method and whose position on the vertical axis represents the balanced accuracy of direct subject-subject transfer using ground truth labels.

SML attempts to infer the balanced accuracy of each individual classifier in the ensemble and weights the classification provided by each subject accordingly. In order to analyze the effectiveness of STIG's ability to infer the accuracy of each ensemble subject from unsupervised data, the learned ensemble weights from each test subject were plotted against the true individual balanced accuracy of each direct subject-to-subject transfer of each subject in the ensemble. As shown in Figure 5, a clear correlation can be seen between the balanced accuracy of the direct subject-subject transfer with the weights assigned according to Equation (8).

### 3.3. Experiment 2: real-time feedback

#### 3.3.1. Paradigm

**Figure 6:**
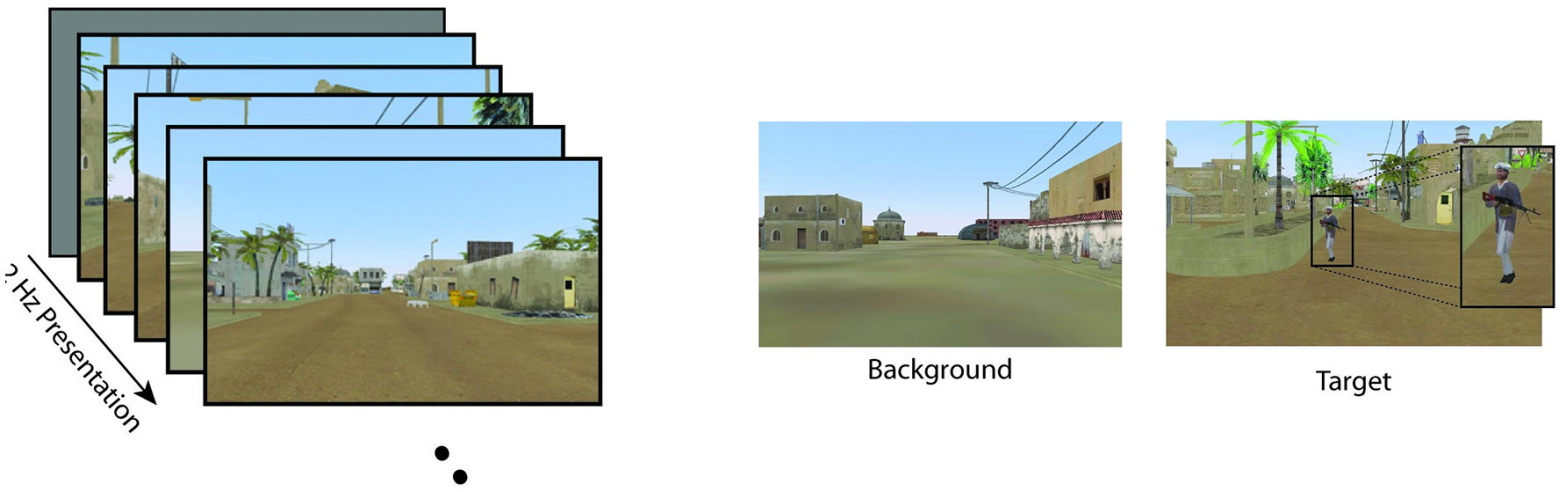
**Visualization of the modified ICT2 paradigm used in the real-time feedback session in Experiment 2**. This modified paradigm replicated the TO condition from ICT where only targets and background images were shown at 2 Hz. Figure reproduced from Marathe et al. (2015) with permission.

The ICT paradigm was modified for the real-time feedback experiment, resulting in the ICT2 paradigm. Subjects participated in a similar RSVP task in which only target and background images were presented (see Figure 6). Images were shown at 2 Hz with no inter-stimulus interval. The ratio of target to background images was 1:9. Subjects sat approximately 60 cm away from a 21′′ LCD monitor which displayed RSVP images that spanned the entire screen. Unlike Experiment 1, subjects in Experiment 2 did not perform any manual button pressing and were instead instructed to inaudibly count the number of targets observed during a single block. Participants conducted 15 blocks of 1 min duration in which 120 images were presented in each block. Participants were given a self-paced rest interval between each block to mitigate any fatigue effects.

**Figure 7:**
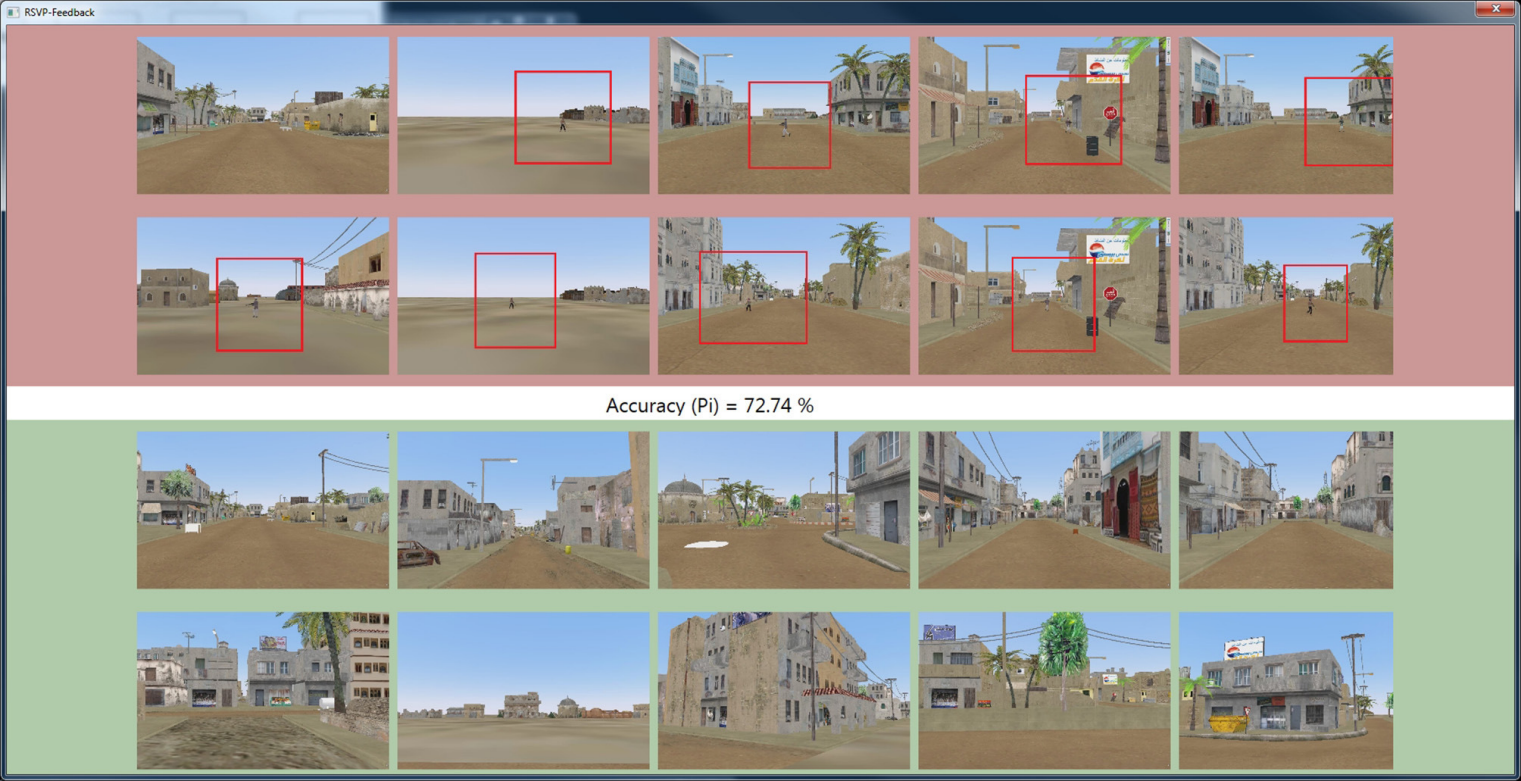
**Screen capture of real-time feedback visualization used in Experiment 2**. As each RSVP image is presented to the subject, the STIG method classifies the trial in real-time, and the results are presented in the above feedback window where target and non-target images, sorted by score, are presented in red and green windows respectively along with balanced accuracy of current block (odd blocks) or combined two previous blocks (even blocks). At the end of each block, the participant was shown the block results pictured here. After image classification, target images appeared on the feedback screen with a red box circumscribing the target in order to facilitate performance assessment of the user.

Each image was classified as target or non-target in real-time from a 1-s epoch of EEG data immediately following the image presentation. A separate 21′′ LCD monitor immediately to the left of the image display monitor presented the feedback results in real-time. Although feedback was continuously displayed, subjects were instructed to only observe the feedback screen during the rest intervals between blocks as to maintain focus on the RSVP task. The feedback window, as shown in Figure 7, displays the 10 targets with the highest score and 10 non-targets with the highest score, where score is the un-signed value of Equation (7). This provided a running measure of how accurately the system classified the images. At the conclusion of each block, the feedback window also displayed balanced accuracy from a single block for odd numbered blocks or the combined accuracy of the last two blocks for even blocks. The feedback window was reset for each odd block.

#### 3.3.2. Data collection

Ten new subjects (9 male, average age 32.7 years) participated in ICT2. EEG data was collected with a BioSemi Active Two system (Amsterdam, Netherlands) at 256 Hz from 64 scalp electrodes arranged in a 10–10 montage and referenced off-line to the average of the left and right mastoids. All signals were digitally filtered from 0.1 to 50 Hz. BCI2000 was used for data collection and on-line classification (Schalk et al., 2004).

#### 3.3.3. Analysis

**Figure 8:**
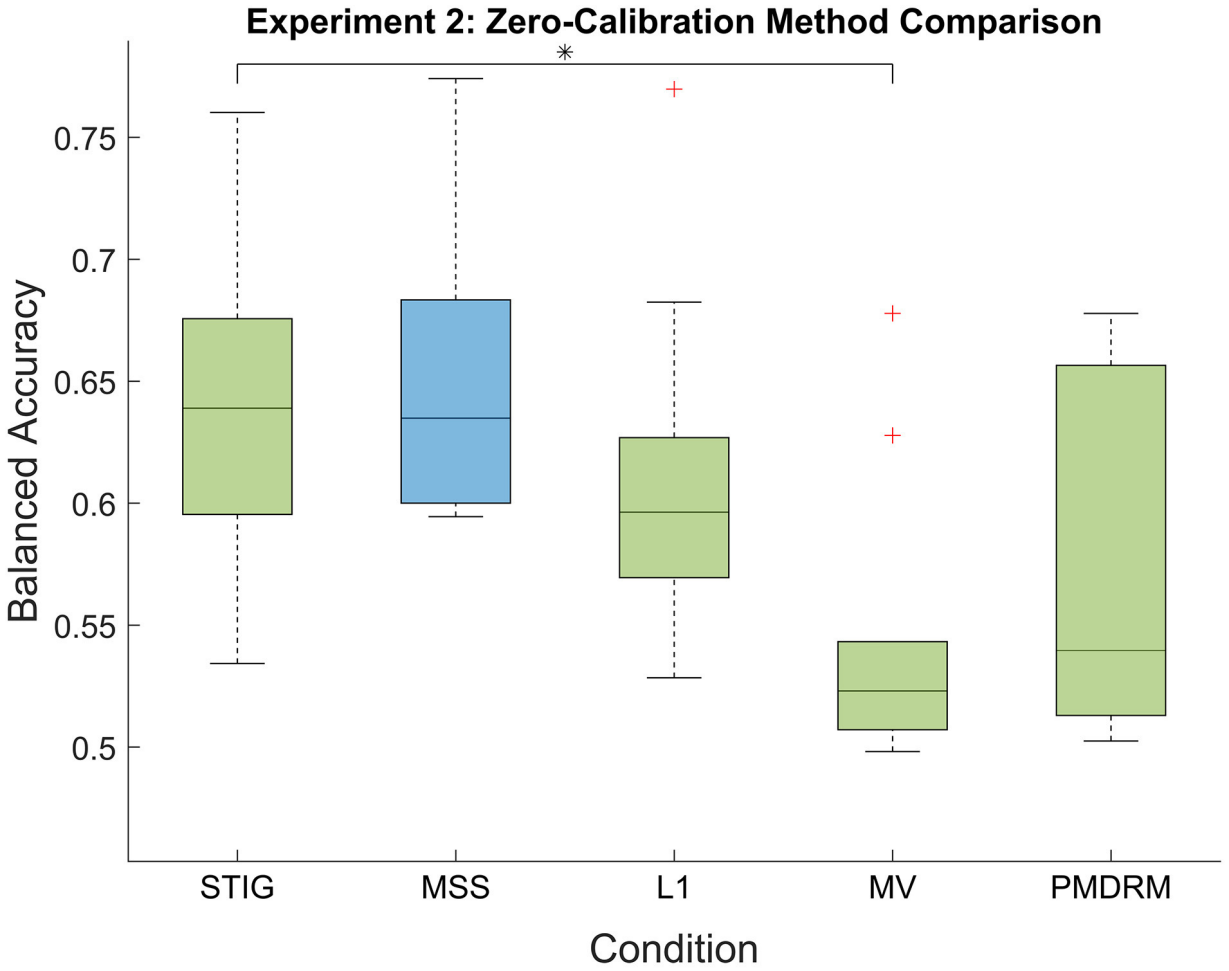
***Post-hoc* balanced accuracy results of the zero-calibration methods for Experiment 2**. Green box plots represent implementable algorithms, while the blue box plots, MSS, represent a method which requires omniscient knowledge. The middle line and boundaries of the box represent median and inner quartile range, the dashed line represents the range and the asterisks indicate statistical significance of pairwise comparisons between STIG and the competing methods. Here, the STIG method significantly outperforms the MV method. The + represents statistical outliers.

**Figure 9:**
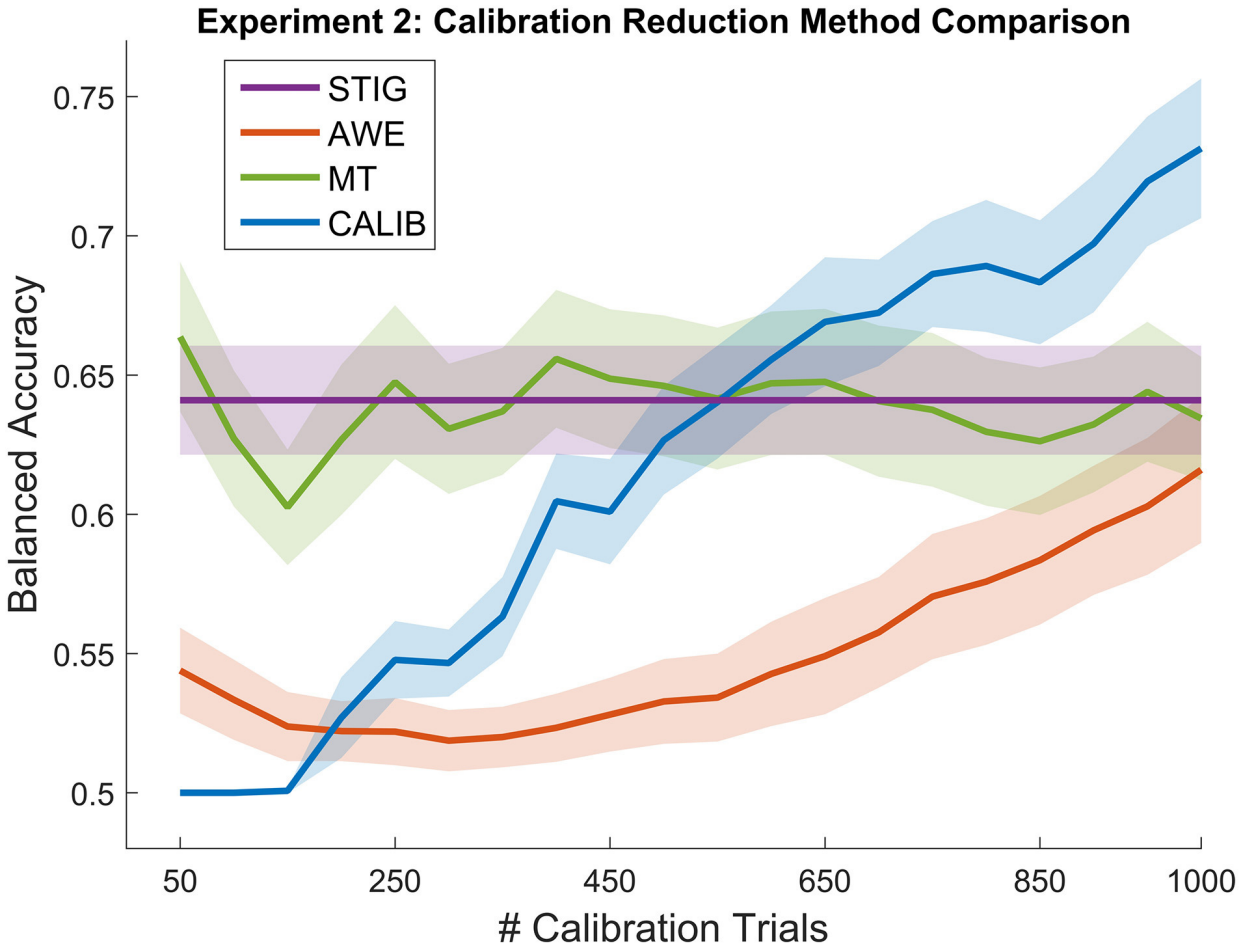
**Balanced Accuracy as a function of number of calibration trials for the calibration reduction methods (AWE and MT) and the traditional within-subject calibration method (CALIB) tested on the real-time feedback paradigm in Experiment 2**. The proposed zero-calibration method STIG, which is independent of the number of calibration trials, is plotted for comparison. Lines represent means averaged over all subjects (*N* = 32) while shaded regions represent standard error.

**Figure 10:**
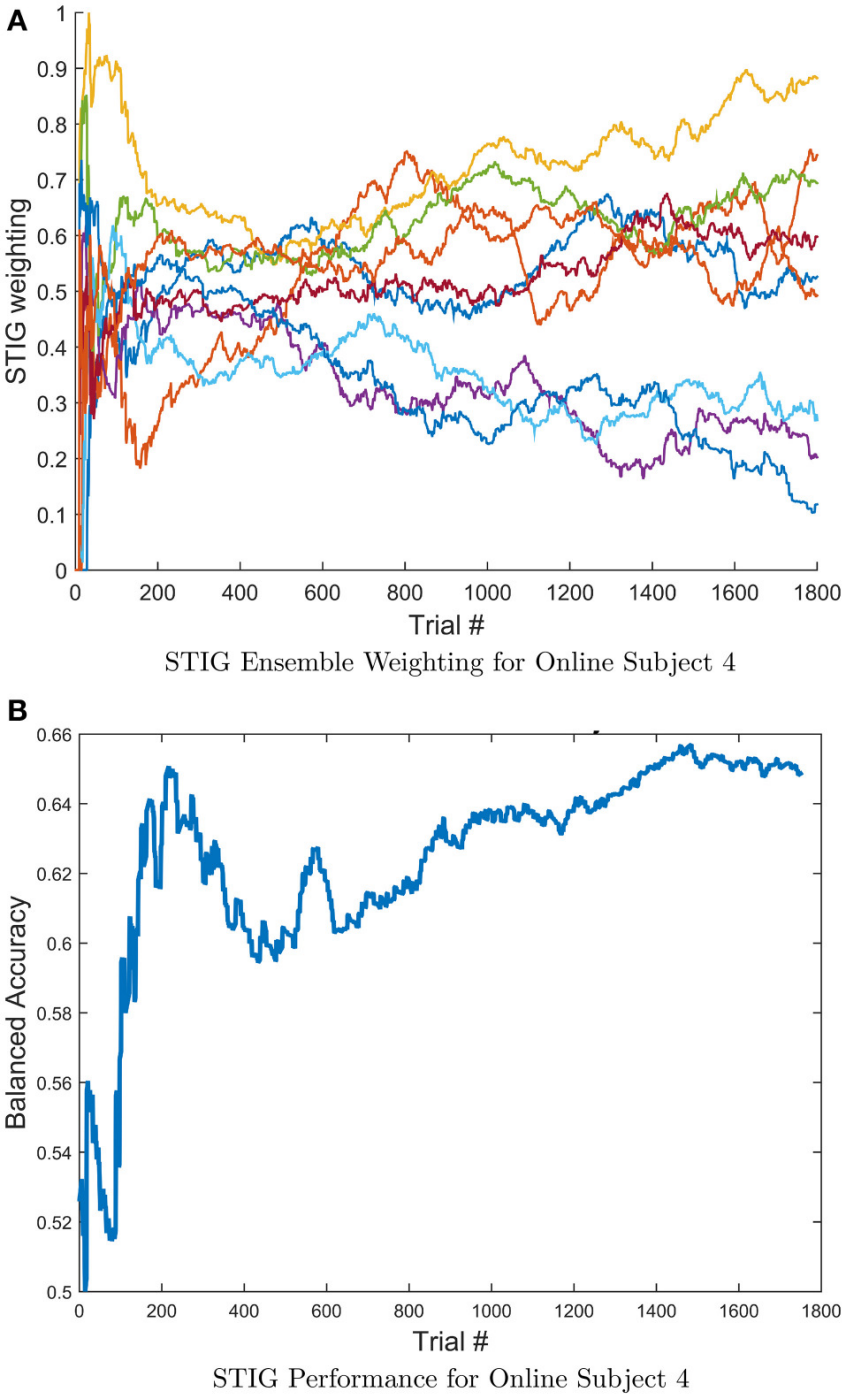
**(A)** Evolution of the STIG ensemble weights for a single subject computed causally throughout Experiment 2. For clarity, only the top 10 ensemble subjects are shown. **(B)** Running estimate of the balanced accuracy for subject 4 over the length of the session. Notice that the ensemble weights vary considerably while the overall STIG performance shows an increasing trend throughout the experiment, indicating an advantage for adaptive methods.

**Figure 11:**
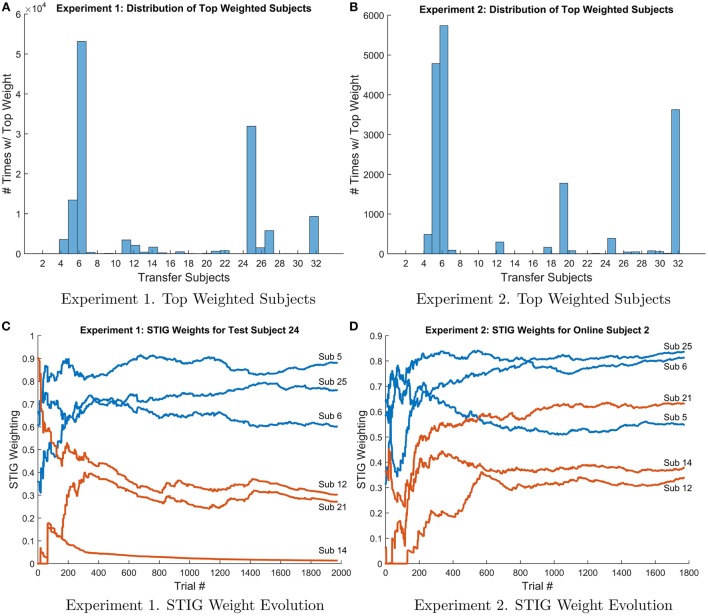
**(A,B)** Histogram of top weighted transfer subject in Experiment 1 (left) and 2 (right). For each test subject and each trial #, the top weighted transfer subject was recorded and the frequency of each ensemble subject selected as the top subject is shown above. **(C,D)** Evolution of STIG weights for subject 24 (Experiment 1, left) and subject 2 (Experiment 2, right) over the course of the experiment. For clarity, only six transfer subjects are shown. The top three weighted transfer subjects (subs 5, 6, and 25) are shown in blue and three low weighted transfer subjects (subs 12, 14, and 21) are shown in red.

**Figure 12:**
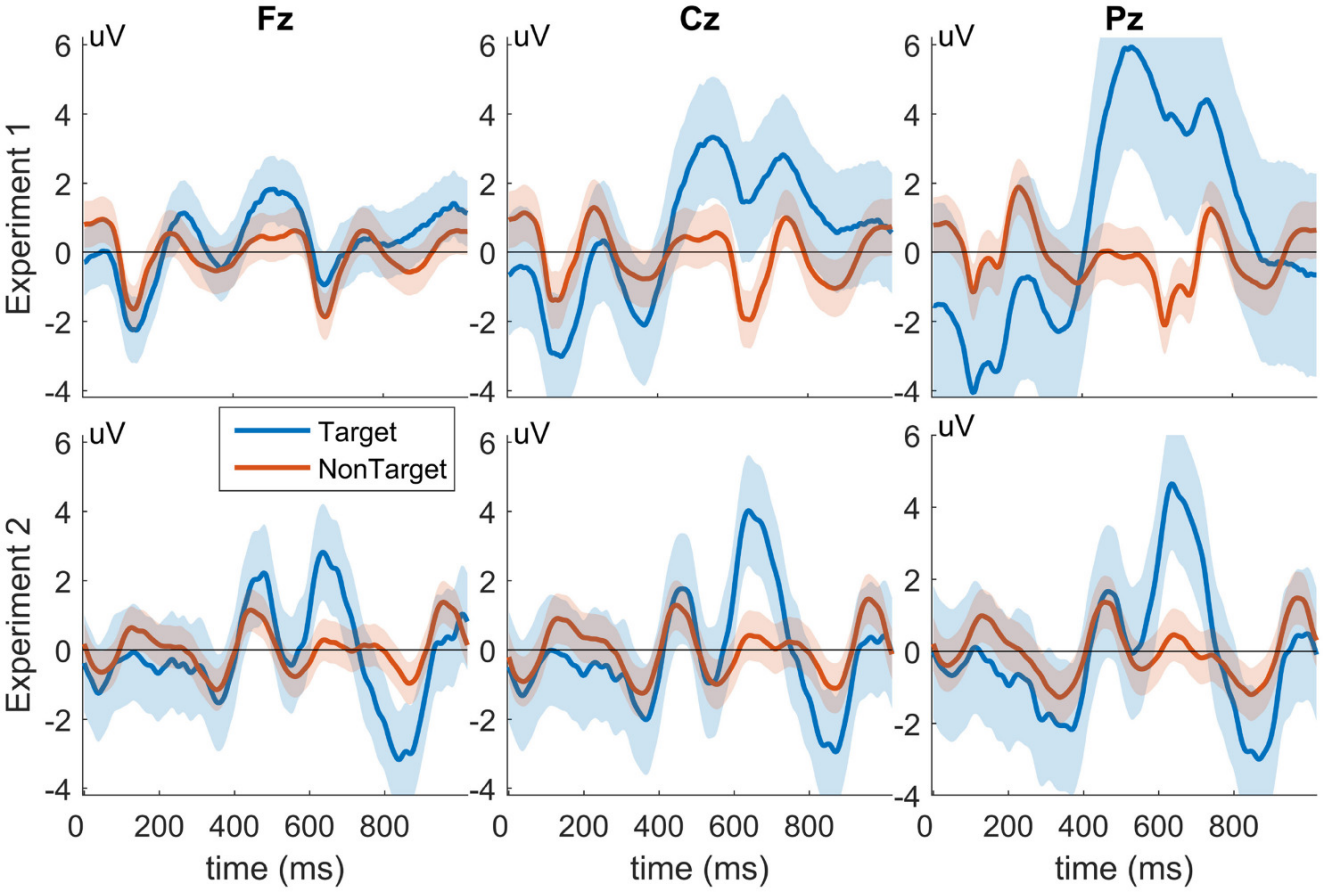
**Grand-Average target and non-target responses from Experiments 1 and 2**. The top three panels show the average responses from Experiment 1 and the bottom three from Experiment 2. Responses from channel Fz, Cz, and Pz are shown (left, middle, and right columns, respectively). Shaded regions represent standard deviation. X-axes show time in *ms*, and y-axes represent signal amplitude in *uV*.

The data was divided into 1 s epochs time-locked to the stimulus onset, [0, 1*s*]. The same 18 channels used in Experiment 1 were also used for classification in Experiment 2. For classification of the online subjects in Experiment 2, MDRM classifiers were generated from all 32 subjects from Experiment 1. Although the MDRM classifiers were trained using data cleaned of artifacts, ICA was not performed on the streaming data from subjects of Experiment 2. For the first 31 trials of the first RSVP block, while $rank\left(\hat{\text{Q}}\right)<32$, MV was used for real-time classification. Only steps (*i*) and (*ii*) of Algorithm 1 were implemented in real-time to classify images and provide feedback. Those results are reported in Table 1. The results reported in Figures 8–**12** were generated in *post-hoc* analysis by implementing steps (*i*)-(*iii*) of Algorithm 1 causally on the streamed data. These *post-hoc* results were compared against the competing methods described in Experiment 1, where for zero-calibaration methods, we compared STIG to MSS, L1, MV, and PMDRM, and for calibration reduction methods, we compared STIG to AWE and MT and CALIB.

**Table 1:** **Total session balanced accuracy by subject from real-time feedback experiment**.

**On-line Feedback Results (Balanced Accuracy)**				
**Subject**	**Mean**	**STD**	**Min**	**Max**
S1	0.54	0.07	0.46	0.68
S2	0.74	0.06	0.60	0.83
S3	0.59	0.05	0.48	0.68
S4	0.64	0.06	0.54	0.78
S5	0.61	0.07	0.47	0.73
S6	0.71	0.07	0.60	0.83
S7	0.62	0.08	0.52	0.81
S8	0.57	0.05	0.51	0.69
S9	0.54	0.06	0.42	0.62
S10	0.67	0.07	0.55	0.76
Overall	0.62	0.06	0.51	0.74

*Shown is the mean accuracy as well as the STD, minimum, and maximum run performance over the 15 online feedback blocks*.

#### 3.3.4. Results

##### 3.3.4.1. Real-time feedback results

Results of the real-time feedback session in Experiment 2 are reported for each subject in Table 1, with an average balanced accuracy of 0.62 ± 0.06. These reflect results from analysis performed during the on-line experiment that was used to generate the real-time RSVP feedback. Two subjects, S2 and S6, perform more than one standard deviation above the mean and two subjects, S1 and S9, perform more than one standard deviation below the mean. Notice that the majority of subjects achieve a maximum online block performance of at least 70% and several subjects are able to achieve online block performances of over 80%.

##### 3.3.4.2. Comparison of zero-calibration algorithms

Figure 8 shows the balanced accuracy results of each zero-calibration method averaged across all subjects in Experiment 2. As with Experiment 1, a one-way ANOVA showed a significant difference between the five methods tested [*F*_(4, 49)_ = 4.92;*p* < 0.01]. Using the Tukey-Kramer method for correction of multiple comparisons, the STIG method (0.64 ± 0.07) achieved statistically similar results to the omniscient MSS classifier (0.65 ± 0.06, *p* = 0.9) and significantly out-performed the MV classifier (0.54 ± 0.06, *p* = 0.012). The STIG method achieves slightly higher performance results compared to the L1 (0.61 ± 0.07) and PMDRM (0.55 ± 0.07) classifiers although these were not found to be significant (*p* = 0.83 and *p* = 0.12, respectively).

##### 3.3.4.3. Comparison of calibration reduction algorithms

Figure 9 shows the results of STIG compared to the calibration reduction methods (AWE and MT) and within-subject performance ceiling (CALIB). Unlike the bootstrap analysis performed in Experiment 1, results for the calibration methods in Experiment 2 were calculated causally. The data was partitioned in sequence into calibration and testing. This was done so that the *post-hoc* analysis would simulate online analysis from the real-time feedback experiment. The calibration reduction methods were tested using varying numbers of calibration trials from 50 to 1000 trials.

Similar to Experiment 1, statistical analysis was performed where one-way ANOVAS were computed for each of the 20 calibration size conditions, the resulting *p*-values were corrected using the false-discovery rate (Benjamini and Yekutieli, 2001). As shown in Figure 9, the STIG method significantly outperforms the AWE method for when 50–600 calibration trials are used for AWE training (*p* < 0.05), and comparable performance is attained when 650 or more calibration trials are used. The STIG method achieves similar performance compared to the MT method (*p* > 0.05) across all numbers of calibration trials used. Unlike the performance shown in Experiment 1 (Figure 3), the MT method does not appear to show a gradual increase in performance with increasing number of calibration trials, as would be expected. Compared to the standard within-subject calibration method (CALIB), STIG achieves significantly higher balanced accuracies when 50–350 calibration trials are available for the CALIB method (*p* < 0.05). For the 400–1000 calibration size conditions, STIG achieves statistically similar performance to the CALIB method (*p* > 0.05).

## 4. STIG characterization

### 4.1. Evolution of SML weights over experiment

To further characterize the transfer properties of the ensemble learner in the STIG method, the ensemble weights from STIG were plotted over time. The estimated weights are tracked as STIG classifies each trial in Experiment 2 causally. Note that in the real-time feedback experiment, as each new trial becomes available, the ensemble classification from that trial gets added to the window of previous classified trials that is used by the STIG algorithm. Thus, STIG updates its ensemble weights for each trial. Figure 10A shows the weights of 10 ensemble subjects for subject 4 as they evolve over the course of the real-time experiment. It is apparent that the relative weight of each subject in the ensemble is not static but rather evolves throughout the experiment, varying by as much as 0.6. Figure 10B illustrates a running estimate of the balanced accuracy for subject 4 where the STIG performance shows an increasing trend over the length of the session.

Histogram plots and weight evolution curves of the top weighted transfer subjects were made and are shown in Figure 11. Figures 11A,B show the distribution of top weighted subjects for experiments 1 and 2 respectively. For each test subject and for each test trial, the ensemble subject with the top weight assigned by the STIG method was recorded and a histogram was made showing the frequency to which each transfer subject was weighted the highest for a given trial. These histogram plots show which subjects are utilized the most in the ensemble transfer. The top weighted subjects from Experiment 1 were subjects 5, 6, 25, and 32, whereas the top weighted subjects from Experiment 2 were subjects 5, 6, 19, and 32. Notice that subjects 5, 6, and 32 are among the top weighted subjects across both experiments. Figures 11C,D show the STIG weights for the top three subjects as well as three lower performing subjects over-time for Experiments 1 and 2, respectively. The top three high-weighted subjects (subjects 5, 6, and 25) are shown in blue and the three low-weighted subjects (12, 14, and 15) are shown in red. Note, only six subjects are shown instead of all 32 for visual clarity. It can be seen in both Figures 11C,D that the STIG weights vary over-time. Additionally, Figure 11D shows a lower-ranked ensemble subject (subject 21) eventually overtaking the higher-ranked ensemble subject (subject 5) at around 600 trials.

## 5. Discussion

The field of transfer learning for BCI is rapidly increasing with a push to develop algorithms that require less and less training data. Although the field has shown more progress in developing algorithms that can reduce calibration, there still remains very little progress on general purpose, zero-calibration algorithms that are capable of classifying brain-signal data from a new subject without any prior training data. This study combines the recent efforts in information geometry and joint classification to present the Spectral Transfer with Information Geometry (STIG) method, an unsupervised ensemble learner that requires zero calibration data to out-perform other state-of-the-art calibration suppression and calibration reduction methods.

The effectiveness of STIG was tested and validated with both off-line and on-line experimentation. For off-line analysis in Experiment 1, STIG significantly outperforms the two competing zero-calibration based ensemble methods (L1 and MV). This demonstrates a benefit gained from a spectral based ensemble learner when applied to BCI transfer. In theory, the SML will provide an ensemble accuracy that is within an additive constant of the best performing subject in the ensemble, and this bound improves with increasing ensemble size. As shown in Figures 2, 8, STIG achieves and exceeds the performance of the best performing classifier in the ensemble. Together, this demonstrates that with neither a calibration session nor any labeled training data, the STIG method should equal the best performing subject in the ensemble and can potentially do better. As compared to the calibration reduction methods for Experiment 1, the STIG method significantly outperforms the AWE method for all numbers of available calibration data and outperforms the MT method and traditional within-subject calibration methods when only a limited amount of training data is available (<400 trials), which approximates to a 5–10 min calibration session. With large amounts of training data available however, the MT and within-subject calibration methods achieve comparable performance to the STIG method. Nevertheless, these methods still require training data and thus necessitate a deliberate calibration session in order to use them. The analyzed data from the real-time paradigm in Experiment 2 show similar conclusions, overall demonstrating that the STIG method has the ability to out-perform other zero-calibration methods and can achieve comparable–and often better–performance to methods which require training data.

More than simply training a single classifier with historical data, STIG provides a unique and adaptable transfer to each test subject. Figure 10 shows for a representative test subject that the relative weighting of each member of the ensemble is not static with time, and, in fact, varies quite significantly throughout the experiment. According to Figure 5, where STIG ensemble weights are shown to be correlated with direct subject-to-subject transfer accuracy, there exist a non-stationarity in the ability of any one ensemble member to classify the test subject. Thus, the adaptive nature of STIG provides a significant advantage over traditional zero-calibration methods which do not adapt after the beginning of the experiment. Figures 11C,D also show that this non-stationarity exists even with subjects that are consistently top-weighed. Despite this non-stationarity, there exist subjects that tend to be consistently top-weighed throughout all test subjects and throughout all trials as seen in Figures 11A,B, where several subjects (notably 5, 6, and 32) have frequency counts that are several orders of magnitude higher than most other subjects. These subjects show high generalizability as their models transfer well to new subjects, achieving high subject-to-subject prediction accuracy, which the STIG method is able to infer.

For on-line implementation, consideration must be given to the computational complexity of the presented algorithm. In the presented experiments, convergence of Algorithm 1 completed well within the half second window before the next EEG epoch was available to classify. Much of the computational work is performed off-line in finding the class means of each of the ensemble subjects. During on-line implementation, Algorithm 1 incurs the following computational costs (*m* classifiers in the ensemble, *T* samples in an EEG epoch, *C* channels used for classification, *n* trials collected): 4*m*(*T*−1)*C*^2^ + O(*mTC*) from feature extraction (2) for each classifier in the ensemble, $\frac{64}{3}mC^{3}+O{\left(mC\right)}^{2}$ from the MDRM classification (5), 2(2*n*−1)*m*^2^+O(*m*) from the covariance estimation in (6), $\frac{1}{3}m^{3}+O\left(m^{2}\right)$ from the eigendecomposition of **Q**, and O(*nm*) for each iteration of (*iii*) in Algorithm 1. The costly steps of the algorithm take place in the extraction and classification of the information geometric feature. The spectral transfer contributes relatively little to the computational complexity. Regardless, it can be further diminished by simply updating the eigendecomposition based on the rank one update of an additional trial without the re-estimation of **Q** (Gu and Eisenstat, 1994). More difficult would be to reduce the cost of the MDRM classification which grows combinatorially with the size of the ensemble, but in practice, this has not proven to be prohibitive for on-line implementation with real-time feedback.

There exists a small difference between the results of the two experiments in which there is a nominal decrease in balanced accuracy from Experiment 2 as compared to Experiment 1. Analysis from Experiment 1 was performed in a similar way most methods use when comparing BCI transfer; using a leave-one-subject-out cross validation transfer where one test subject is extracted from a dataset and the remaining subjects are used as training for BCI transfer. However, leave-one-out cross validation is not indicative of a use-case for practical BCI transfer where data from the same experiment is used for transfer. Experiment 2 attempts to more realistically capture real world use with cross-experiment transfer by using data from Experiment 1 as historical training data to classify subject data in Experiment 2. However, Experiment 2 contains several confounding factors which might explain its decreased performance. Experiment 2, which uses data from Experiment 1 for transfer, contains several paradigmatic differences that can negatively impact the performance of this cross-experiment transfer. First, Experiment 1 displays target, non-target, and distractor images to each subject, whereas Experiment 2 only utilizes target and non-target images. Additionally, subjects in Experiment 1 performed manual button pressing during target images, while subjects in Experiment 2 did not. Finally, Experiment 1 was collected using e-prime software and Experiment 2 was collected using BCI2000, which may have different latencies between the two systems. All of these factors likely contribute to observed differences seen in target response amplitude and latency between the two experiments (Figure 12), which shows the grand-average target and non-target responses for Experiments 1 (top) and 2 (bottom) for channels Fz, Cz, and Pz. These morphological differences in target waveforms can negatively impact the performance of the cross experiment transfer and potentially explain the decrease in accuracy from Experiment 1 to Experiment 2.

The STIG method has an initial start-up cost associated with it as it requires at least *m* predictions from each of its *m* ensemble classifiers in order to estimate the covariance matrix and generate accurate ensemble weights. As a result, during the beginning of online classification of a new subject, STIG resorts to simple majority voting until *m* trials have been collected. Additional work will seek to alleviate this issue and allow the STIG method to be applied immediately from the start, for example, one approach could be to use the L1-regularization technique from Fazli et al. (2011) as a warm-start for the STIG weight estimation. Additionally, the STIG method utilizes all previous test data labels for ensemble weight estimation, however, this may not be optimal during long test sessions as information from ensemble predictions from the beginning of a session may not be useful during the end of a session, due to fatigue effects and other sources of non-stationarity (i.e., an ensemble subject that transfers well during one portion of a test session may not transfer well during another portion). One possible solution to this issue would be to utilize an iterative covariance matrix update method together with a decay-rate to put emphasis on more recent classification predictions. These current limitations of the proposed method will be addressed in future work which will potentially improve the transfer capabilities of the STIG method.

## 6. Conclusion

This paper presents a novel method, termed Spectral Transfer with Information Geometry (STIG), to achieve unsupervised BCI transfer learning that outperforms the state-of-the-art calibration reduction and suppression methods when little or no calibration data is present. Additionally, this paper also validates the STIG method for use in real-time feedback BCI systems, representing a step-forward in the overall goal of attaining a general purpose, user-independent BCI system.

## Author contributions

NW, VL, AB, and BL designed research; NW, VL, AB, and KB performed research; NW, VL, and AB analyzed data and NW, VL, AB wrote the paper.

### Conflict of interest statement

The authors declare that the research was conducted in the absence of any commercial or financial relationships that could be construed as a potential conflict of interest.
